# Investigating the Impact of Reproductive Coercion and Intimate Partner Violence on Psychological and Sexual Wellbeing

**DOI:** 10.1177/08862605241253026

**Published:** 2024-05-16

**Authors:** Nicola Sheeran, Alisha Jenkins, Tiffany Humphreys, Sonja Ter Horst, Mary Higgins

**Affiliations:** 1 School of Applied Psychology, Griffith University, Brisbane, QLD, Australia

**Keywords:** reproductive coercion and abuse, intimate partner violence, psychological health, sexual health, violence against women

## Abstract

Emerging research suggests that reproductive coercion and abuse (RCA), like intimate partner violence (IPV), is associated with poorer mental and sexual health outcomes, including greater symptoms of post-traumatic stress disorder (PTSD) and depression and poorer markers of physical and sexual health such as sexually transmitted infections, unplanned pregnancies and lowered sexual agency. Although victims/survivors of RCA report long-lasting impacts on future relationships, including fear and anxiety, little is known about impacts of RCA on anxiety and general wellbeing, nor emotional and mental components of sexual health that comprise a person’s sexual self-concept. With community samples of participants in Australia, we conducted two studies to explore the impact of RCA and IPV on psychological (study 1) and sexual (study 2) health outcomes. Study 1 (*n* = 368) found that experiencing IPV and RCA both significantly and uniquely contributed to poorer mental health outcomes. After controlling for age and IPV, RCA significantly predicted symptoms of depression, anxiety, stress, PTSD, and reduced satisfaction with life. Study 2 (*n* = 329) found that IPV and RCA differentially predicted various components of sexual health. IPV predicted decreased sexual satisfaction and increased sexual anxiety, depression, and fear of sexual encounters. After controlling for age and IPV, RCA significantly and uniquely predicted lower levels of sexual assertiveness and increased sexual depression and fear of sexual encounters, but not sexual satisfaction or anxiety. We conclude that RCA is associated with significant psychological distress and a negative sexual self-concept that may impact future relationships. Screening for both IPV and RCA across settings is warranted.

## Introduction

Reproductive coercion and abuse (RCA) is an understudied form of violence against women, distinct in its intent to interfere with another’s reproductive autonomy (Miller et al., 2010). RCA can include the use of physical, psychological, sexual, economic, and other strategies to coerce or force a person with female reproductive capacity to become pregnant; continue a pregnancy; terminate a pregnancy; take/use contraception; or, perpetrators may interfere with contraception to cause pregnancy. Most research has focused on intimate partner RCA, where heterosexual ciswomen experience RCA perpetrated by their current or past male intimate partner. However, non-male partners, family members, health professionals, and religious leaders can also be perpetrators or instigators (Moulton et al., 2021; Rowlands & Walker, 2019). There is ongoing definitional debate around whether structural forms of RCA (i.e., via laws, policies, and social norms) should also be considered (DeJoy, 2019); however, in this article, we focus on interpersonal forms of RCA.

To date, inconsistencies in RCA measurement and lack of conceptual clarity have plagued much of the research on RCA. Most research has utilized the reproductive coercion (RC) scale originally developed by Miller et al. (2010) and subsequently adapted to suit specific contexts, often reducing the scale to four items. However, this scale only measures pregnancy coercion and contraceptive sabotage and does not measure pregnancy-preventing RCA, such as threats and/or violence to ensure pregnancy is terminated/miscarried or forced contraceptive use (Sheeran et al., 2022; Tarzia & Heggarty, 2021). A recent study demonstrated that pregnancy-promoting RCA and pregnancy-preventing RCA were experienced at roughly equal rates (7.9% pregnancy-promoting; 9.4% pregnancy preventing; Sheeran et al., 2022), yet the prevalence and impacts of pregnancy-preventing RCA are largely unknown. RCA intersects with intimate partner violence (IPV), domestic and family violence (DFV), and sexual violence (SV), arguably differentiated by the intent of the perpetrator (Tarzia & Hegarty, 2021). Yet a lack of conceptual clarity has led to conflations of SV and RCA (i.e., “stealthing”), which impacts prevalence rates, particularly for younger populations (Tarzia & Hegarty, 2021). Thus, accurate prevalence rates have been difficult to ascertain with recent research on lifetime prevalence in help-seeking populations in Australia ranging from 2.3% to 5.9% (Cheng et al., 2021; Price et al., 2022) and up to 15% to 17% (Galrao et al., 2022; Sheeran et al., 2022) depending on the setting and measurement. International studies suggest setting dependent prevalence rates of 5% to 40% (Rowlands & Walker, 2019).

Studies investigating risk factors for RCA have been similarly hampered by measurement and definitional inconsistencies, resulting in mixed findings for age, race, and relationship status. For example, some studies find younger age (Katz et al., 2017), being separated/single (Price et al., 2022), and from a culturally and linguistically diverse background (Grace & Anderson, 2018) are risk factors, and others do not (Sheeran et al., 2022). However, other forms of violence do appear to be significantly associated with RCA (Price et al., 2022). RCA has also been associated with lethal IPV violence (Bagwell-Gray et al., 2021; Grace et al., 2022), and IPV may increase the likelihood of RCA (Price et al., 2022). However, RCA also appears to occur independently of other forms of abuse, with around 10% of those reporting RCA reporting no IPV (Alexander et al., 2021).

### RCA and IPV Psychological Outcomes

IPV has consistently been shown to impact negatively on psychological health, with individuals who have experienced IPV reporting poorer mental health compared to those who have not experienced IPV (Lagdon et al., 2014). Being a victim/survivor of IPV is associated with higher rates of depression (Devries et al., 2013; Do et al., 2013; Kamimura et al., 2016), post-traumatic stress disorder (PTSD), and anxiety (Do et al., 2013), with severity of the violence a critical moderator (Lagdon et al., 2014). Research investigating the association between RCA and psychological outcomes is in its infancy but suggests that RCA similarly has a negative impact on psychological health, with several studies establishing associations between pregnancy-promoting RCA and PTSD and depression (Alexander et al., 2021; Anderson et al., 2017; Capasso et al., 2019; Grace et al., 2022; Jiwatram-Negron et al., 2022; McCauley et al., 2014). RCA and IPV uniquely contribute to psychological difficulties (Alexander et al., 2021). Thus, emerging evidence suggests that RCA impacts depression and PTSD symptoms, though associations with other mental health difficulties, such as anxiety, stress, and general wellbeing, have not yet been established. Relatively less is known about the sexual health impacts of RCA.

### RCA and IPV Sexual Health Outcomes

Sexual health has been defined as “a state of physical, emotional, mental, and social wellbeing in relation to sexuality; it is not merely an absence of disease, dysfunction or infirmity” (World Health Organization, 2006) and is comprised of a range of components including physical (e.g., unintended pregnancy, sexually transmitted infections [STI]), mental/emotional/social wellbeing in relation to sexuality (e.g., sexual anxiety, depression, fear); sexual self-efficacy; sexual agency/assertiveness; sexual satisfaction; and an absence of violence/coercion (Smylie et al., 2013).

Perhaps unsurprisingly, IPV has been shown to negatively impact sexual health, including reduced sexual agency and ability to negotiate condom use, which increases risk of HIV, STIs, and unplanned pregnancy (Bagwell-Gray, 2019; Bergmann & Stockman, 2015; Seth et al., 2015; Swan & O’Connell, 2012; Yoshioka et al., 2020). Women in violent relationships reported being assertive and seeking sexual health did not outweigh the costs of putting their physical safety in jeopardy (Bagwell-Gray, 2019). Katz et al. (2017) explored the impact of RCA and IPV on sexual and contraception self-efficacy and contraception use and found that women with a history of RCA reported lower contraceptive and sexual self-efficacy and a significantly reduced rate of contraceptive use during last vaginal sex. Capasso et al. (2019) found that women experiencing pregnancy coercion were 78% more likely to test positive for an STI, worried more about contracting HIV, and were more fearful of negotiating condom use than those not experiencing pregnancy coercion. Thus, IPV and pregnancy-promoting RCA appear to have similar negative impacts on physical and sexual health, agency, and assertiveness. Emerging qualitative evidence suggests RCA may also impact mental/emotional components of sexual health.

Mitchell and Bennett (2020) conducted a qualitative study with Fijian university students on abortion coercion and sexual relationships and found long-term impacts, including apprehension about dating, trust issues, and an inability to open up emotionally to potential partners followed them into future relationships (Mitchell & Bennett, 2020; Lévesque et al., 2021) qualitative study explored the impact of RCA on the psychological, sexual/reproductive, and relational wellbeing of 21 female victims/survivors. Participants emphasized the psychological impacts of RCA, such as fear, shame, depression, anxiety, nightmares, flashbacks, and social isolation, and impacts on sexual wellbeing, including fears relating to STIs, lower sexual desire and pleasure, and negative desires toward having a child, with impacts on sexual wellbeing seen as longer lasting (Lévesque et al., 2021).

### The Current Study

As highlighted above, an emerging body of evidence suggests that, like IPV, pregnancy-promoting RCA significantly impacts psychological and sexual health. However, much of the literature above has utilized insufficient measures of RCA. Further, most studies have been undertaken with niche samples, including those accessing family planning and counseling for unplanned pregnancy, with relatively less known about RCA, its intersections with IPV, and psychological and sexual health outcomes in the general community. Thus, the overarching aim of the current studies was to explore the impact of RCA on psychological and sexual health outcomes using a community sampling strategy and a measure of RCA that included pregnancy-preventing behaviors. We report the findings of two studies: the first focused on psychological outcomes, and the second focused on sexual health outcomes.

## Study 1

The aim of the first study was to explore the impact of RCA on psychological outcomes. As previously highlighted, emerging evidence suggests that RCA is associated with greater symptoms of depression and PTSD (Alexander et al., 2021; Capasso et al., 2019; Grace et al., 2022; Jiwatram-Negron, et al., 2022; McCauley et al., 2014). However, this research has only considered pregnancy coercion and/or contraceptive sabotage. Forced abortion and other forms of pregnancy-preventing RCA are arguably as likely to impact mental health outcomes and wellbeing, yet no research has explored this. Prior research has also focused on specific populations, such as specific racial/cultural backgrounds (Alexander et al., 2021; Capasso et al., 2019; McCauley et al., 2014), women accessing health services (Anderson et al., 2021; Price et al., 2019; Jiwatram-Negron et al., 2022), or college-aged women (Grace et al., 2022). So, while there is some evidence to suggest that RCA is associated with depression and PTSD, little is known about broader psychological outcomes for victims/survivors of RCA in the community. Thus, the current study explored whether RCA and/or IPV predicted poorer mental health outcomes. We hypothesized that IPV and RCA would independently predict poorer mental health, including increased symptoms of depression, anxiety, stress, and PTSD and decreased satisfaction with life. We also tentatively hypothesized (in line with Alexander et al.,’s [2021] findings) that RCA would significantly predict mental health outcomes after controlling for IPV.

## Method

### Participants

Three hundred and sixty-eight participants were recruited via convenience sampling as part of a larger study. Participants had to be female, over the age of 16, residing in Australia, English-speaking, have been in at least one relationship since age 16, and have engaged in sexual intercourse with another person. The samples were well educated with years of education ranging from 11 to 20 years (*M* = 15.18, *SD* = 2.46). Age ranged from 18 to 62 years (*M* = 27.79, *SD* = 10.17). Demographics are provided in Table 1.

**Table 1.** Demographic Sample Characteristics for Study 1 Exploring Associations Between RCA and IPV on Mental Health (*n* = 368).

Variable	Percentage of Sample %	*N*
Current relationship status		
Single	21.3	77
Married/Defacto	43.9	162
In a relationship	32.1	118
Separated/divorced	2.4	9
Gender identity		
Woman	99	364
Nonbinary	1	4
Sexual identity		
Heterosexual	81.4	300
Bisexual	15.3	56
Lesbian/Gay/Queer	2.5	9
Pansexual	0.8	3
Parent		
Yes	21.7	80
Employment status		
Full-time	18.2	69
Part-time/casual	49.7	183
Student	26.4	97
Not employed	5.7	21
Ethnicity		
White	87.2	321
Asian	3.8	14
Middle Eastern	1.1	4
Hispanic	1.1	4
First Australian	3.3	12
Mixed/Other	3.6	13
Type of IPV experienced*		
Any form of IPV	69.8	257
Physical abuse	40.5	149
Emotional abuse	62.5	230
Harassment	35.1	129
Sexual abuse	11.4	42
Any IPV with no RCA	48.1	177
No IPV and no RCA	30.1	111
Type of RCA^#^		
Any form of RCA	21.7	80
Pregnancy preventing	5.8	21
Pregnancy promoting	18.7	69
Both pregnancy preventing and promoting	2.1	8
Any RCA with no IPV	2.7	10
Any RCA and any IPV	19.1	70

*Note.* RCA = reproductive coercion and abuse; IPV = intimate partner violence.

* IPV by a current or former partner in the past 12 months.

# RCA in their lifetime.

### Materials

Demographics collected included gender, sexual orientation, age, birth country, postcode, ethnicity, religion, annual household income, education, employment, relationship, and parental status.

RCA was measured using a revised Reproductive Coercion Scale (Miller et al., 2010). The original 11-item scale consisted of six items measuring pregnancy coercion and five items investigating birth control sabotage. Additional items, “made you hide birth control because you were afraid they would get upset with you for using it” and “made you terminate a pregnancy you wanted to continue with” were added. The items were preceded with the stem “Has someone you were dating or going out with ever. . .” Each question was answered yes or no, and endorsement of any item indicated the presence of RCA. This scale has demonstrated consistent moderate internal validity (α = .66 to .76; Grace & Anderson, 2018); α = .84 in the current study.

IPV was measured using the Community Composite Abuse Scale (CCAS; Loxton et al., 2013). The 28-item scale includes items assessing physical abuse (10 items), emotional abuse (13 items), and harassment (4 items) perpetrated by a current or past partner. A single item assessed sexual assault (“forced me to take part in unwanted sexual activity”). Participants were asked whether they had experienced behaviors such as that their partner “told me I wasn’t good enough” (emotional abuse), “kicked/bit/hit with a fist” (physical abuse), “harassed me at work” (harassment) using a binary (yes/no) format. The scale also assesses the severity of the abuse by rating the frequency with which they experienced any behaviors in the past 12 months, using a Likert scale ranging from 1 = *only once* to 5 = *daily* and providing not applicable and not in the last 12 months options (scored as 0). Participants were classified as having experienced IPV if they had experienced at least one IPV behavior in the past 12 months. The severity data for the total scale and each sub-scale was averaged. The CCAS has demonstrated high internal validity (α > .85; Loxton et al., 2013); α = .89 in the current study.

Psychological distress was measured using the 21-item Depression Anxiety Stress Scale (DASS-21; Lovibond & Lovibond, 1993), which forms three subscales, depression, anxiety, and stress, with seven items measuring each. Example items include “I couldn’t seem to experience any positive feeling at all” (depression), “I was aware of dryness of my mouth” (anxiety), and “I found it hard to wind down” (stress). Participants rated how often each had applied to them in the past week on a 4-point scale from 0 = “Did not apply” to 3 = “Applied to me very much.” Sub-scale scores were summed, then doubled, and ranged from 0 to 42, which allows normative interpretations to be made. Raw scores can be converted to clinical ranges that indicate whether symptoms are considered normal, mild, moderate, severe, or extremely severe. The measure has good reliability and validity (Lovibond & Lovibond, 1993), with α = .90, .87, and .89 for the depression, anxiety, and stress subscales, respectively, in the current study.

PTSD was measured using the Impact of Event Scale-Revised (IES-R). The IES-R consists of 22 items assessing the subjective distress (intrusion, avoidance, and hyperarousal) resulting from traumatic events (Weiss & Mamar, 1997). Participants were asked to specify a stressful life event and when this incident occurred. Fifty percent of the sample reported having experienced a traumatic event, with sexual abuse/assault (36.6%), death or suicide of a friend or loved one (22.68%), physical abuse (14.43%), forced abortion (4.1%), car accidents (3%) and natural disasters (3%) commonly reported. Participants then indicated whether they had ever experienced difficulties such as “I thought about it when I didn’t mean to,” and how distressed or bothered they ever were by them on a 5-point scale ranging from 0 (*not at all*) to 4 (*extremely*), yielding a total score ranging from 0 to 88, with higher scores representing higher levels of distress. The measure has excellent convergent validity and internal reliability (α = .96; Creamer et al., 2003); α = .96 in the current study.

General wellbeing was measured using the Satisfaction with Life Scale (SWLS: Diener et al., 1985). The SWLS consists of 5 items, including “In most ways my life is close to my ideal,” which are rated on a Likert-style scale ranging from 1 (*strongly disagree*) to 7 (*strongly agree*), with possible total scores ranging from 5 to 35 and higher scores indicating greater satisfaction with life. The SWLS has demonstrated good internal validity (α = .79 to .95; Pavot & Diener, 2008), α = .91 in the current study.

### Procedure

Ethical approval was granted by Griffith University Human Research Ethics Committee (HREC; Ref No: 2020/067), and all participants provided informed consent. Participants were recruited from the community using convenience sampling methods, including the university psychology first-year subject pool, flyers, social media, and email and could elect to win one of six $50 prize vouchers or course credit. The online survey (hosted on Limesurvey) advised participants that they would be asked about personal and sensitive information regarding their relationships, and participants rated their level of distress on a 10-point scale at the beginning, middle, and end of the survey. Participants with ratings of 8 or above were redirected to a list of helplines and were precluded from continuing the survey.

## Results

Data was analyzed using IBM SPSS 29. Fifteen participants were identified as outliers, predominantly on the IPV severity measures, having experienced frequent IPV. As removal did not change the significance or interpretation of the analyses, they were retained. Zero-order correlations were calculated for all variables (see Table 2). The predictors IPV and RCA had significant positive associations with SWL, Depression, Anxiety, Stress, and PTSD. Age was also significantly correlated with four of the five dependent variables and was included in subsequent analyses. Five hierarchical regressions were used to test the relationship between IPV and RCA in the prediction of depression, anxiety, stress, PTSD, and SWL, controlling for age (see Table 3).

**Table 2.** Means, Standard Deviations and Intercorrelations Between Age, RCA, IPV, and Mental Health Outcomes.

Variables	1	2	3	4	5	6	7	8
1. Age	—							
2. RCA	.068	—						
3. IPV	−.008	.240**	—					
4. Depression	−.250**	.147**	.217**	—				
5. Anxiety	−.273**	.174**	.185**	.662**	—			
6. Stress	−.165**	.195**	.148**	.735**	.771**	—		
7. PTSD	−.141*	.175*	.189**	.279**	.359**	.383**	—	
8. SWL	.076	−.133*	−.238**	−.559**	−.413**	−.420**	−.167*	—
*M*	27.79	.22	.161	10.89	8.79	14.70	44.14	24.32
*SD*	10.167	.413	.393	10.21	8.97	10.07	24.08	6.97

*Note.* IPV = intimate partner violence; RCA = reproductive coercion and abuse; PTSD = post-traumatic stress disorder; SWL = satisfaction with life.

* *p* < .05, **p < .01.

**Table 3.** Hierarchical Multiple Regression of Age, IPV and RCA on Depression, Anxiety, Stress, Post-traumatic Stress Disorder (PTSD), and Satisfaction with Life.

Variables	Model 1					Model 2					Model 3				
	*B*	*SE*	*β*	*sr* ^2^	95% CI	*B*	*SE*	*β*	*sr* ^2^	95% CI	*B*	*SE*	*β*	*sr* ^2^	95% CI
Depression															
Age	−.25***	0.05	−.25	−.25	[−.35, −.15]	−0.25***	0.05	−.25	−.25	[−.35, −.15]	−0.26***	0.05	−.26	−.26	[−.36, −.16]
IPV						5.59***	1.29	.22	.22	[3.07, 8.12]	4.86***	1.32	.19	.18	[2.27, 7.45]
RCA											2.88***	1.26	.12	.11	[.4, 5.36]
∆*R*^2^						.046***					.013*				
* R* ^2^	.063***					.109					.122				
Anxiety															
Age	−0.24***	0.05	−.27	−.27	[−.33, −.15]	−0.24***	0.04	−.27	−.27	[−.33, −.15]	−0.25***	0.04	−.28	−.28	[−.33, −.16]
IPV						4.18***	1.13	.18	.18	[1.96, 6.4]	3.32**	1.15	.15	.14	[1.06, 5.58]
RCA											3.39**	1.1	.16	.15	[1.22, 5.55]
∆*R*^2^						.034***					.023**				
* R* ^2^	.074***					.108					.131				
Stress															
Age	−0.16***	0.05	−.17	−.17	[−.27, −.06]	−0.16***	0.05	−.16	−.16	[−.26, −.06]	−0.17***	0.05	−.18	−.18	[−.27, −.08]
IPV						3.78**	1.31	.15	.15	[1.2, 6.35]	2.65*	1.33	.1	.1	[.04, 5.27]
RCA											4.42***	1.27	.18	.18	[1.91, 6.92]
∆*R*^2^						.022**					.031***				
* R* ^2^	.027**					.049					.08				
PTSD															
Age	−0.34*	0.17	−.14	−.14	[−.67, −.01]	−0.29	0.17	−.12	−.12	[−.62, −.04]	−0.31	0.17	−.13	−.13	[−.64, −.02]
IPV						9.19*	3.67	.18	.17	[1.95, 16.42]	7.36	3.75	.14	.14	[−.03, 14.75]
RCA											7.66*	3.76	.15	.14	[.25, 15.07]
∆*R*^2^						.03*					.02*				
* R* ^2^	.02*					.05					.07				
Satisfaction with life															
Age	0.05	0.04	.08	.08	[−.02, .12]	0.05	0.04	.08	.08	[−.02, .12]	0.05	0.04	.08	.08	[−.01, .12]
IPV						4.21***	0.9	−.24	−.24	[−5.98, −2.44]	−3.84***	0.93	−.22	−.21	[−5.66, −2.02]
RCA											−1.45	0.89	−.09	−.08	[−1.19, .29]
∆*R*^2^						.052***					.007				
* R* ^2^	.006					.062					.069				

*Note.* IPV = intimate partner violence; RCA = reproductive coercion and abuse; CI = confidence intervals.

* *p* < .05, **p < .01, ***p < .001.

Hierarchical regression was used to test the relationship between age, IPV, and RCA in the prediction of depression. Age was entered in the first step and was significant *F*(1,365) = 24.436, *p* < .001, accounting for 6.3% of variance. Older participants were less likely to experience depression than younger ones. IPV was entered in the second step, and *F* change indicated a significant improvement in prediction over age alone, *F*(2,364) = 22.288, *p* < .001. IPV accounted for 4.6% of variance, with those who had experienced IPV reporting higher depression than those who had not. RCA was entered in the third step and made a significant contribution to the prediction of depression, *F*(3, 363) = 16.770, *p* < .001, accounting for an additional 1.3% of variance. After controlling for age and IPV, participants who had experienced RCA reported higher depression than those who had not experienced RCA. RCA, age, and IPV each significantly contributed to our prediction of depression.

Hierarchical regression was used to test the relationship between age, IPV, and RCA in the prediction of anxiety. Age was entered in the first step and was significant *F*(1, 365) = 29.357, *p* < .001, accounting for 7.4% of variance. Older participants were less likely to experience anxiety than younger ones. IPV was entered in the second step, and *F* change indicated a significant improvement in prediction over age alone, *F*(2, 364) = 22.045, *p* < .001. IPV accounted for 3.4% of variance, with those who had experienced IPV reporting higher anxiety than those who had not. RCA was entered in the third step and made a significant contribution to the prediction of anxiety, *F*(3, 363) = 18.190, *p* < .001, accounting for an additional 2.3% of variance. After controlling for age and IPV, participants who had experienced RCA reported higher anxiety than those who had not experienced RCA. RCA, age, and IPV each significantly contributed to our prediction of anxiety.

Hierarchical regression was used to test the relationship between age, IPV, and RCA in the prediction of stress. Age was entered in the first step and was significant *F*(1, 365) = 10.251, *p* = .001, accounting for 2.7% of variance. Older participants were less likely to experience stress than younger ones. IPV was entered in the second step and *F* change indicated a significant improvement in prediction over age alone, *F*(2, 364) = 9.387, *p* < .001. IPV accounted for 2.2% of variance, with those who had experienced IPV reporting higher stress than those who had not. RCA was entered in the third step and made a significant contribution to the prediction of stress, *F*(3, 363) = 10.462, *p* < .001, accounting for an additional 3.1% of variance. After controlling for age and IPV, participants who had experienced RCA reported higher stress than those who had not experienced RCA. RCA, age, and IPV each significantly contributed to our prediction of stress.

Hierarchical regression was used to test the relationship between age, IPV, and RCA in the prediction of PTSD. Age was entered in the first step and was significant *F*(1, 198) = 4.004, *p* = .047, accounting for 2% of variance. Older participants were less likely to experience PTSD than younger ones. IPV was entered in the second step, and *F* change indicated a significant improvement in prediction over age alone, *F*(2, 197) = 5.192, *p* = .006. IPV accounted for 3% of the variance, with those who had experienced IPV reporting higher PTSD than those who had not. RCA was entered in the third step and made a significant contribution to the prediction of PTSD, *F*(3, 196) = 4.902, *p* = .003, accounting for an additional 2% of variance. After controlling for age and IPV, participants who had experienced RCA reported higher PTSD than those who had not experienced RCA. RCA, age, and IPV each significantly contributed to our prediction of PTSD.

Hierarchical regression was used to test the relationship between age, IPV, and RCA in the prediction of SWL. Age was entered in the first step and was not significant, *F*(1, 365) = 2.138, *p* = NS. IPV was entered in the second step, and *F* change indicated a significant improvement in prediction, *F*(2, 364) = 12.086, *p* < .001. IPV accounted for 5.6% of the variance, with those who had experienced IPV reporting lower SWL than those who had not. RCA was entered in the third step and made a significant contribution to the prediction of SWL, *F*(3, 363) = 8.981, *p* < .001, accounting for an additional 0.7% of variance. After controlling for age and IPV, participants who had experienced RCA reported lower SWL than those who had not experienced RCA. RCA and IPV both significantly contributed to our prediction of SWL, while age did not.

## Discussion

The current study’s aim was to explore the impact of RCA and IPV on psychological outcomes. As hypothesized, we found that experiencing IPV and RCA both significantly and uniquely contributed to poorer mental health outcomes. After controlling for age and IPV, RCA significantly predicted symptoms of depression, anxiety, stress, PTSD, and reduced satisfaction with life. Our findings mirror those of Alexander et al. (2021), who found RCA and IPV increased the risk of PTSD and depression. Our study extends these findings, showing RCA is also associated with increased stress, anxiety, and decreased satisfaction with life, demonstrating how extensively this form of violence impacts mental health in the general community. Further, we found the effect of RCA on stress was greater than the effect of IPV, suggesting that this specific form of violence increases psychological stress, which in turn can impact physical health (Cohen, 2007). Further research is required to understand why.

Compared to other studies, our sample had a greater proportion of participants who had experienced IPV only (Grace & Anderson, 2018) and fewer who had experienced RCA only. For example, Alexander et al. (2021), in their sample of Black/African American women, found nearly 10% had experienced RCA only, while we found only 2%. Of note, Alexander et al. (2021) only measured pregnancy coercion and contraceptive sabotage and not pregnancy-preventing RCA, such as forced/pressured abortion. We found that nearly 3% of our sample had experienced forced abortion as the only form of RCA in their relationship. A recent study suggested rates of pregnancy-preventing RCA were marginally higher than pregnancy-promoting RCA (9.4% vs. 7.9%; Sheeran et al., 2022). However, in our study, pregnancy-promoting RCA was far more common (18.7%) than pregnancy preventing (5.8%). This is likely due to our use of a community sample rather than samples of help-seeking pregnant people. Concerningly, nearly 70% of our sample had experienced IPV, with emotional abuse being most common. Recent research has shown that emotional abuse is associated with worse mental and physical health than other forms of abuse (i.e., physical; Scott et al., 2023). Our findings demonstrate the importance of assessing multiple facets of IPV and RCA in mental health settings. Finally, our sample included a higher proportion of individuals identifying as bisexual than the general population, likely due to the age of our sample as the highest rate of bisexual identification is in 25–39-year-olds (Parliament of Australia, 2022). There is a dearth of research investigating RCA in LGBTIQA+ communities making it difficult to know how our sample characteristics may have impacted our findings, though further research is warranted.

## Study 2

The aim of study two was to explore the relationship between IPV and RCA on sexual health outcomes. Emerging evidence suggests that IPV and RCA are associated with poorer sexual health outcomes. However, most studies have focused on physical sexual health outcomes, including unintended pregnancies, STI risks, and ambivalence toward pregnancies/contraception (Bagwell-Gray, 2019; Bergmann & Stockman, 2015; Katz et al., 2017; Seth et al., 2015; Swan et al., 2012; Yoshioka et al., 2020). Less is known about how RCA impacts positive and negative indices of a woman’s sexual self-concept, such as sexual satisfaction and sexual assertiveness (positive indices of sexual self-concept) and sexual anxiety, depression, and fear (negative indices of sexual self-concept; Hoi Nga Ng et al., 2022). Qualitative studies have suggested that RCA is associated with lower sexual assertiveness and higher sexual anxiety, depression, and fear of sexual relationships (Lévesque & Rousseau, 2019; Mitchell & Bennett, 2020). However, it is not known whether IPV and RCA differentially impact these components of sexual health. The current study explored whether RCA and/or IPV predicted poorer sexual health outcomes. Given the dearth of research, we tentatively hypothesized that IPV and RCA would independently predict poorer sexual health including reduced sexual satisfaction and assertiveness and increased sexual depression, anxiety, and fear of sexual relationships. We tentatively hypothesized that RCA would significantly predict sexual health outcomes after controlling for IPV.

## Method

### Participants

Three hundred and twenty-nine participants were recruited via convenience sampling. Participants had to be aged between 17 and 50 years, residing in Australia, English-speaking, a biological and heterosexual female with a current or previous sexual partner, who had not yet experienced menopause or commenced hormone replacement therapy due to the decline in sexual activity and satisfaction commonly associated with these factors (Monteleone et al., 2018). Participants ages ranged from 17 to 48 years (*M* = 23.08, *SD* = 7.21). Demographics are provided in Table 4.

**Table 4.** Demographic Sample Characteristics for Study 2 Exploring Associations Between RCA and IPV on Sexual Health (*n* = 329).

Variable	Percentage of Sample %	*n*
Current relationship status		
Single	40.7	134
Married/Defacto	27.4	90
In a relationship	29.1	96
Separated/divorced	1.2	4
Parent		
Yes	14.4	47
Education		
Year 12 (or equivalent)	58.3	192
Certificate/Diploma/Advanced diploma	22.4	74
Graduate diploma/Bachelor	10.6	35
Postgraduate degree	3.9	13
Employment status		
Full-time	11.7	38
Part-time/casual	70	230
Student	17.6	58
Not employed	0.9	3
Ethnicity		
White	72.2	238
Asian	10.3	34
First Australian	2.8	9
African	1.9	6
Middle Eastern	1.7	6
Pacific Islander	1.7	6
Mixed/Other	9.1	30
Type of IPV experienced		
Any form of IPV	55.6	183
Physical abuse	47.4	156
Psychological abuse	50.5	166
Harassment	35.1	115
Sexual abuse	19	63
Any IPV with no RCA	55.6	133
No IPV and no RCA	40.4	184
Type of RCA		
Any form of RCA	20.5	68
Pregnancy preventing	5.4	18
Pregnancy promoting	20.5	68
Both preventing and promoting	5.4	18
Any RCA with no IPV	3.6	12
Any RCA and any IPV	20.8	69

*Note.* IPV and RCA by a current or former partner. IPV = intimate partner violence; RCA = reproductive coercion and abuse.

## Materials

Demographics collected included age, birth country, postcode, ethnicity, religion, annual household income, education, employment, relationship, and parental status.

RCA was measured using the 13-item Reproductive Coercion Scale used in study one with two additional items added to strengthen the measurement of pregnancy-preventing RCA, “Has your partner ever threatened to end the relationship if you did not terminate a pregnancy?” and “Has your partner ever hurt or threatened to hurt you while you were pregnant because you did not agree to terminate a pregnancy?” Cronbach’s alpha was .81 in the current study.

IPV was measured using the Composite Abuse Scale-Revised-Short-Form (CASr-SF; Ford-Gilboe et al., 2016). The 16-item scale includes items assessing physical abuse (6 items), psychological abuse (8 items), and sexual abuse (2 items) perpetrated by a current or past partner. Participants were asked whether they had experienced a range of behaviors such as that their partner “shook, pushed, grabbed or threw me” (physical abuse), “blamed me for their violent behavior” (psychological abuse), or “forced me or tried to force me to have sex” (sexual abuse) using a 6-point Likert scale ranging from 0 (never) to 5 (daily/almost daily). Participants were classified as having experienced IPV if they had ever experienced any of these behaviors. A total score was created by computing the mean of all 16 items (range = 0–80). The scale has high internal validity (α = .94; Ford-Gilboe et al., 2016; α = .95 current study).

Sexual satisfaction was measured using the 25-item Index of Sexual Satisfaction (ISS: Hudson et al., 1981). Items including “my partner does not satisfy me sexually” were answered on a 7-point Likert scale ranging from 0 (none of the time) to 6 (all of the time). Items were summed, subtracting the number of completed scores, multiplying this figure by 100, and dividing the number of items completed by 6, creating a total score ranging from 0 to 100. Scores above 30 indicate a clinically significant problem in sexual satisfaction, while scores above 70 indicate severe stress and possible violence in the relationship. In the current study, all participants were above 30 but below 70. The ISS shows high internal validity (α = .93; Hudson et al., 1981; α = 91 current study).

The Multidimensional Sexuality Questionnaire (MSQ; Snell et al., 1993) is a 60-item scale assessing 12 aspects of sexuality, each 5 items. Sexual assertiveness (e.g., “I am very assertive about the sexual aspects of my life”), sexual anxiety (e.g., “I am worried about the sexual aspects of my life”), sexual depression (e.g., “I feel discouraged about my sex life”), and fear of sexual encounters (e.g., “I sometimes have a fear of sexual relationships”) subscales were used and measured on a 5-point Likert scale from 0 (not at all characteristic of me) to 4 (very characteristic of me). Items were summed with higher scores indicating greater amounts of that aspect of sexuality. The scale has good internal validity (α = .77, .83, .82, and .92 for sexual assertiveness, sexual anxiety, fear of sexual encounters, and sexual depression, respectively; Snell et al., 1993; α = .79, .86, and .88 current study). Fear of sexual encounters was poor (α = .41) but improved to α = .84 after deleting “I am not afraid to be sexually active.”

### Procedure

Ethical clearance was granted by Griffith University Human Research Ethics Committee (Ref No: 2022/406), and all participants provided informed consent. Participants were recruited from the community using convenience sampling methods, including the university psychology first-year subject pool, flyers, social media, and email, and could elect to win one of three $50 prize vouchers or course credit. The online survey (hosted on Qualtrics) advised participants that they would be asked about personal and sensitive information regarding their relationships and sexual experiences, and participants rated their level of distress on a 10-point scale at the middle and end of the survey. Participants with ratings of 8 or above were redirected to a list of helplines and were precluded from continuing the survey.

## Results

Data was analyzed using SPSS 29. Thirteen participants were identified as outliers on the IPV severity measure, having experienced frequent IPV. As this was a focal variable, these participants were retained. Zero-order correlations were calculated for all study measures (see Table 5). IPV was significantly associated with sexual satisfaction, anxiety, depression, and fear of sexual encounters. RCA was significantly associated with sexual anxiety, assertiveness, depression, and fear of sexual encounters. Age was also significantly correlated with two of the five dependent variables and was included in subsequent models. Five hierarchical regressions were used to test the relationship between IPV and RCA in the prediction of sexual satisfaction, sexual assertiveness, sexual anxiety, sexual depression, and fear of sexual encounters, controlling for age (see Table 6).

**Table 5.** Means, Standard Deviations and Intercorrelations Between Age, IPV, RCA, and Sexual Health Outcomes.

	1	2	3	4	5	6	7	8
1. Age	—							
2. IPV	.131*	—						
3. RCA	.133*	.399**	—					
4. Sexual satisfaction	−.019	.200**	.057	—				
5. Sexual anxiety	−.113*	.183**	.143**	.157**	—			
6. Sexual assertiveness	.059	−.086	−.171**	.048	−.425**	—		
7. Sexual depression	.036	.252**	.216**	.208**	.760**	−.369**	—	
8. Fear of sexual encounters	−.190**	.139*	.153**	.103	.656**	−.466**	.473**	—
*M*	23.08	8.92	.21	50.71	10.30	14.78	9.85	13.44
*SD*	7.21	14.31	.407	7.82	4.55	4.55	4.77	3.89

*Note.* IPV = intimate partner violence; RCA = reproductive coercion and abuse.

* p < .05, **p < .001

**Table 6.** Hierarchical Multiple Regression of Age, IPV, and RCA on Sexual Satisfaction, Assertiveness, Depression, Anxiety, and Fear of Sexual Encounters.

Variables	Model 1					Model 2					Model 3				
	*B*	*SE*	*β*	*sr* ^2^	95% CI	*B*	*SE*	*β*	*sr* ^2^	95% CI	*B*	*SE*	*β*	*sr* ^2^	95% CI
Sexual satisfaction															
Age	−.02	.06	−.02	.00	[−.02, .09]	−.05	.06	−.05	.002	[−.17, .06]	−.05	.06	−.04	.002	[−.16, .06]
IPV						.12***	.03	.22	4.7	[.06, .18]	.13***	.03	.23	.045	[.06, .19]
RCA											−.69	1.14	−.03	.10	[−2.94, 1.55]
∆*R*^2^						.047***					.001				
* R* ^2^	.000					.048					.049				
Sexual assertiveness															
Age	.03	.03	.05	.056	[−.03, .10]	.04	.03	.07	.068	[−.02, .11]	.05	.03	.08	.081	[−.02, .12]
IPV						−.03	.02	−.09	−.092	[−.06, .005]	−.01	.02	−.03	−.028	[−.05, .03]
RCA											−1.80**	.67	−.16	−.147	[−3.12, −.49]
∆*R*^2^						.008					.022**				
* R* ^2^	.003					.012					.033				
Sexual depression															
Age	.02	.03	.03	.03	[−.04, .09]	−.002	.03	−.003	−.003	[−.07, .06]	−.009	.03	−.01	−.013	[−.08, .06]
IPV						.09***	.02	.27	.270	[.05, .13]	.07***	.02	.22	.204	[.03, .11]
RCA											1.51*	.68	.13	.117	[−.16, 2.85]
∆*R*^2^						.073**					.014*				
* R* ^2^	.001					.074					.088				
Sexual anxiety															
Age	−.08*	.04	−.13	−.128	[−.15, −.013]	−.10**	.04	−.16	−.155	[−.17, −.03]	−.11**	.04	−.16	−.161	[−.18, −.04]
IPV						.07***	.02	.20	.199	[.03, .10]	.06***	.02	.17	155	[.02, .10]
RCA											.94	.69	.08	.074	[−.41, 2.30]
∆*R*^2^						.039***					.005				
* R* ^2^	.016					.04					.061				
Fear of sexual encounters															
Age	−.10***	.03	−.19	−.191	[−.16, −.04]	−.11***	.03	−.21	−.216	[−.17, −.06]	−.12***	.03	−.22	−.224	[−.17, −.06]
IPV						.05***	.01	.19	.191	[.02, .08]	.04*	.01	.15	.135	[.009, .07]
RCA											1.1*	.55	.11	.105	[.008, 2.19]
∆*R*^2^						.036***					.011*				
* R* ^2^	.036					.073					.084				

*Note.* IPV = intimate partner violence; RCA = reproductive coercion and abuse; CI = confidence interval.

* *p* < .05, **p < .01, ***p < .001.

Hierarchical regression was used to test the relationship between age, IPV, and RCA in the prediction of sexual satisfaction. Age was entered in the first step and was not significant, *F*(1, 327) = .111, *p* = .739. IPV was entered in the second step, making a significant contribution to the prediction of sexual satisfaction *F*(1, 326) = 16.24, *p* < .001. IPV accounted for 4.7% of variance, with those who had experienced IPV reporting greater problems in their sexual satisfaction. RCA was entered in the third step and did not significantly improve prediction over IPV alone, *F*(1, 325) = .373, *p* = .542.

Hierarchical regression was used to test the relationship between age, IPV, and RCA in the prediction of sexual assertiveness. Age was entered in the first step and was not significant, *F*(1, 327) = 1.02, *p* = .311. IPV was entered in the second step and did not make a statistically significant contribution to the prediction of sexual assertiveness *F*(1, 326) = 2.80, *p* = .095. RCA was entered in the third step and made a significant contribution to the prediction of sexual assertiveness *F*(1, 325) = 7.28, *p* = .007. RCA accounted for 2.1% of the variation, with those who had experienced RCA reporting lower sexual assertiveness.

Hierarchical regression was used to test the relationship between age, IPV, and RCA in the prediction of sexual anxiety. Age was entered in the first step and was significant, *F*(1, 327) = 5.48, *p* = .02, accounting for 1.6% of variance. These results indicate that as age increased, the levels of sexual anxiety experienced decreased. IPV was entered in the second step, and *F* change indicated a significant improvement in prediction over the use of age alone *F*(1, 326) = 13.66, *p* < .001. IPV accounted for an additional 4% of the variance, with those who had experienced IPV reporting higher sexual anxiety than those who had not experienced IPV. RCA was entered in the third step and did not make a significant improvement in prediction over the use of age and IPV *F*(1, 325) = 1.90, *p* = .172.

Hierarchical regression was used to test the relationship between age, IPV, and RCA in the prediction of sexual depression. Age was entered in the first step and was not significant, *F*(1, 327) = .414, *p* = .520. IPV was entered in the second step, and *F* change indicated a significant improvement in prediction over the use of age alone *F*(1, 326) = 25.75, *p* < .001. IPV accounted for 7.2% of the variance, with those who had experienced IPV reporting greater sexual depression than those who had not experienced IPV. RCA was entered in the third step and made a significant contribution to the prediction of sexual depression *F*(1, 325) = 4.87, *p* = .028. RCA accounted for an additional 1.4% of the variance, with those who had experienced RCA reporting greater sexual depression.

Hierarchical regression was used to test the relationship between age, IPV, and RCA in the prediction of fear of sexual encounters. Age was entered in the first step and was significant *F*(1, 327) = 12.33, *p* < .001, accounting for 3.6% of variance. As age increased, fear of sexual encounters decreased. IPV was entered in the second step, and *F* change indicated a significant improvement in prediction over the use of age alone *F*(1, 326) = 12.78, *p* < .001. IPV accounted for 3.6% of the variance, with those who had experienced IPV reporting greater fear of sexual encounters than those who had not experienced IPV. RCA was entered in the third step and made a significant contribution to the prediction of fear of sexual encounters *F*(1, 325) = 3.92, *p* = .04. RCA accounted for an additional 1.1% of the variance with those who had experienced RCA reporting greater fear than those who had not. RCA, age, and IPV each significantly contributed to our prediction of fear of sexual encounters.

## Discussion

The aim of this study was to explore the impact of RCA and IPV on sexual health, specifically, the positive and negative indices of sexual self-concept. We found that IPV and RCA differentially predicted various components of sexual health. IPV predicted decreased sexual satisfaction and increased sexual anxiety, depression, and fear of sexual encounters, with greatest impacts on negative indices of sexual self-concept, a finding consistent with having experienced sexual abuse (Hoi Nga Ng et al., 2022). Conversely, RCA had the greatest impact on positive indices of sexual self-concept, significantly and uniquely predicting lower levels of sexual assertiveness. RCA also predicted increased sexual depression and fear of sexual encounters but not sexual satisfaction or anxiety.

Several studies have found that those who experience RCA report lower efficacy/assertiveness in negotiating contraception and greater difficulties communicating sexual choices (Swan et al., 2012; Yoshioka et al., 2022). Our study replicates and extends these findings by demonstrating that sexual assertiveness generally is impacted when someone has experienced RCA, likely because the ability of victims/survivors to be assertive may be hindered by the power imbalance often present in their relationship, as well as fear for their own safety (Bagwell-Gray, 2019). Consistent with this, experiences of RCA predicted greater fear of sexual encounters. Previous qualitative research has suggested that RCA leads to greater apprehension about dating (Mitchell & Bennett, 2020), greater suspicion of new partners, and fear of revictimization (Levesque et al., 2021), which is in line with our findings suggesting significant fear of sexual encounters is ongoing. Like study 1, we found that pregnancy-promoting RCA was significantly more common than pregnancy-preventing RCA in our sample (20% vs. 5%), with around 20% having experienced any form of RCA. Further, over 50% of our sample had experienced IPV, with psychological IPV again the most common. Our findings show the diverse and unique impact RCA can have on a woman’s sexual self-concept and ability to be assertive in the sexual domain.

## General Discussion

The overarching aim of the current studies was to explore the impact of RCA on psychological and sexual health outcomes using a community sampling strategy and a measure of RCA that included pregnancy-preventing behaviors, such as forced/coerced abortion. We found that IPV and RCA had significant effects on people’s psychological and sexual wellbeing. After controlling for age and IPV, RCA significantly predicted increased depression, anxiety, stress, PTSD, sexual depression, fear of sexual encounters, and reduced satisfaction with life and sexual assertiveness. Our findings join an emerging body of research showing that RCA over and above IPV has wide-ranging psychological impacts for victims/survivors (Alexander et al., 2021). Our study was the first to consider the impact of RCA on the components of sexual wellbeing comprising a person’s sexual self-concept, which is an understudied but critical area associated with overall wellbeing (Anderson, 2013), with most studies having focused on physical indices, such as risk of STI’s and unintended pregnancy. We found that sexual assertiveness was especially impacted by RCA and not IPV. Our findings also lend support to qualitative studies that found long-lasting impacts on future relationships by showing that RCA is associated with fear of sexual encounters and sexual depression.

While our findings demonstrate that RCA uniquely impacts mental and sexual health for women in the community who may or may not be seeking help from services, less is known about why and how. RCA encompasses a broad range of behaviors that, in part, overlap with IPV, SV, family violence, and coercive control while also having components that are unique. The current study goes some way to demonstrating that RCA is an important form of violence to understand and assess, given the breadth of mental and sexual health issues that result. However, more research is needed to look at whether specific forms of RCA and specific forms of IPV co-occur and subsequently, whether they differentially impact mental and physical health. Severity is also an important moderator of IPV and psychological outcomes that is unable to be considered with current RCA measurement tools.

Our use of a community sampling strategy and measurement of pregnancy-preventing RCA contributes to our understanding of the prevalence of the various forms of RCA. We found that pregnancy-promoting RCA was more common than pregnancy-preventing RCA in our community sample. This is much lower than in help-seeking populations (see Sheeran et al., 2022). In line with previous studies, we found that a small proportion of participants experience RCA without IPV and report both forms of RCA. In study 2, all who experienced pregnancy-preventing RCA had also experienced pregnancy-promoting RCA, raising questions as to the temporality of the various behaviors used by perpetrators. Unfortunately, our measure of RCA did not allow us to disentangle who the perpetrator/s was/were, and our sample was not sufficient to undertake detailed analysis of outcomes associated with the different forms of RCA or IPV, a suggested direction for future research.

### Practical Implications

Our findings suggest the need to assess IPV and RCA across mental and sexual health settings, especially given that the different forms of violence are associated with somewhat different outcomes. Routine screening of RCA is seen as highly acceptable to patients, facilitating access to assistance and support (Galrao et al., 2022). Services supporting victims/survivors also need to assess both sexual and psychological health outcomes broadly, as our findings demonstrate the breadth of symptoms experienced. Psychologists, counselors, and those working in mental health need to ask about abuse as it relates to symptom presentations and to have mechanisms in place to ensure confidentiality and comfort for disclosures to occur, as well as clear referral pathways in place.

### Limitations and Future Directions

While our study improved on much of the previous research by including items that assessed pregnancy-preventing RCA, we still only included one and three items in study 1 and 2, respectively, which did not comprehensively assess RCA as we currently understand it (Sheeran et al., 2022; Tarzia & Hegarty, 2021). Major shifts in our understanding of RCA have occurred since the construct’s initial conceptualization by Miller in 2010, yet measurement has not kept pace. Future research is needed to develop a measure of RCA, that comprehensively assesses RCA, moves away from presence/absence, considers severity and intent, and behaviors used by different perpetrators, over time.

Another limitation of our studies was our use of a cross-sectional design, coupled with our measurement of lifetime RCA and lack of measurement of engagement in help-seeking behaviors/whether they had sought treatment. Thus, we were unable to explore temporal associations between the experiences of RCA and psychological and/or sexual health nor establish causation while potentially explaining the small amount of variance explained by our models. Future longitudinal research is needed to explore the complexities of violence and mental/sexual health outcomes over time.

A strength of the study was our inclusion of those with diverse sexual identities and genders in study 1; a limitation in many other studies and in our study 2. While our samples achieved representativeness in some areas, overall, our samples were more educated and less representative of some ethnicities. Our requirement for English language proficiency also means that we were not representative of those with low-English proficiency, a focus for future research (Tarzia et al., 2022).

## Conclusion

Our studies were the first to explore the impact of RCA and IPV in a community sample on a broad range of psychological and sexual health outcomes, including anxiety, stress, satisfaction with life, and a person’s sexual self-concept. Our findings suggest that RCA is associated with significantly poorer psychological health, low sexual assertiveness, and a negative sexual self-concept after controlling for age and IPV. Services supporting victims/survivors need to broadly assess impacts on sexual and psychological health, and those working in sexual and mental health settings need to assess for both IPV and RCA that may be contributing to client’s presentations.

## References

[bibr1-08862605241253026] Alexander K. A. Willie T. C. McDonald-Mosley R. Campbell J. C. Miller E. Decker M. R. (2021). Associations between reproductive coercion, partner violence, and mental health symptoms among young Black women in Baltimore, Maryland. Journal of Interpersonal Violence, 36(17–18), NP9839–NP9863. 10.1177/0886260519860900 PMC6954344 31296104

[bibr2-08862605241253026] Anderson J. C. Grace K. T. Miller E. (2017). Reproductive coercion among women living with HIV: An unexplored risk factor for negative sexual and mental health outcomes. AIDS, 31(16), 2261. 10.1097/QAD.0000000000001620 28832408 PMC5633520

[bibr3-08862605241253026] Anderson R. (2013). Positive sexuality and its impact on overall well-being. Bundesgesundheitsblatt, 56, 208–214. 10.1007/s00103-012-1607-z 23361205

[bibr4-08862605241253026] Bagwell-Gray M. E. (2019). Women’s healing journey from intimate partner violence: Establishing positive sexuality. Qualitative Health Research, 29(6), 779–795. 10.1177/1049732318804302 30371140

[bibr5-08862605241253026] Bagwell-Gray M. E. Thaller J. Messing J. T. Durfee A. (2021). Women’s reproductive coercion and pregnancy avoidance: Associations with homicide risk, sexual violence, and religious abuse. Violence Against Women, 27(12–13), 2294–2312. 10.1177/10778012211005566 34165023

[bibr6-08862605241253026] Bergmann J. N. Stockman J. K. (2015). How does intimate partner violence affect condom and oral contraceptive use in the United States? A systematic review of the literature. Contraception, 91(6), 438–455. 10.1016/j.contraception.2015.02.009 25708504 PMC4442065

[bibr7-08862605241253026] Capasso A. DiClemente R. J. Wingood G. M. (2019). Pregnancy coercion as a risk factor for HIV and other sexually transmitted infections among young African American women. Journal of Acquired Immune Deficiency Syndromes, 82(2), 155–161. 10.1097/QAI.0000000000002174 PMC6820702 31658204

[bibr8-08862605241253026] Cheng Y. Wilson E. G. Botfield J. R. Boerma C. J. Estoesta J. Peters L. J. McGeechan K. (2021). Outcomes of routine screening for reproductive coercion in a family planning service. Sexual Health, 18(5), 349–357. 10.1071/SH21079 34606741

[bibr9-08862605241253026] Cohen S. (2007). Psychological stress and disease. JAMA, 298(14), 1685–1687. 10.1001/jama.298.14.1685 17925521

[bibr10-08862605241253026] Creamer M. Bell R. Failla S. (2003). Psychometric properties of the impact of event Scale-Revised. Behaviour Research and Therapy, 41(12), 1489–1496. 10.1016/j.brat.2003.07.010 14705607

[bibr11-08862605241253026] DeJoy G. (2019). State reproductive coercion as structural violence. Columbia Social Work Review, 17(1), 36–53. 10.7916/cswr.v17i1.1827

[bibr12-08862605241253026] Devries K. M. Mak J. Y. Bacchus L. J. Child J. C. Falder G. Petzold M. Astbury J. Watts C. H. (2013). Intimate partner violence and incident depressive symptoms and suicide attempts: A systematic review of longitudinal studies. PLoS Medicine, 10(5), e1001439. 10.1371/journal.pmed.1001439 PMC3646718 23671407

[bibr13-08862605241253026] Diener E. Emmons R. A. Larsen R. J. Griffin S. (1985). The satisfaction with life scale. Journal of Personality Assessment, 49(1), 71–75. 16367493 10.1207/s15327752jpa4901_13

[bibr14-08862605241253026] Do K. N. Weiss B. Pollack A. (2013). Cultural beliefs, intimate partner violence, and mental health functioning among Vietnamese women. International Perspectives in Psychology, 2(3), 149–163. 10.1037/ipp0000004 PMC3866026 24358448

[bibr15-08862605241253026] Ford-Gilboe M. Wathen C. N. Varcoe C. MacMillan H. L. Scott-Storey K. Mantler T. Hegarty K. Perrin N. (2016). Development of a brief measure of intimate partner violence experiences: The composite abuse scale (revised)-short form (CASR-SF). BMJ Open, 6(12), e012824. 10.1136/bmjopen-2016-012824 PMC5168640 27927659

[bibr16-08862605241253026] Galrao M. Creagh A. Douglas R. Smith S. Brooker C. (2022). Experience of introducing screening for intimate partner violence and reproductive coercion in an urban sexual health clinic. Australian and New Zealand Journal of Public Health, 46, 889–895. 10.1111/1753-6405.13301 36121262

[bibr17-08862605241253026] Grace K. T. Perrin N. A. Clough A. Miller E. Glass N. E. (2022). Correlates of reproductive coercion among college women in abusive relationships: Baseline data from the college safety study. Journal of American College Health, 70(4), 1204–1211. 10.1080/07448481.2020.1790570 32672505 PMC7885792

[bibr18-08862605241253026] Grace K. T. Anderson J. C. (2018). Reproductive coercion: A systematic review. Trauma, Violence & Abuse, 19(4), 371–390. https://doi.org/10.1177/1524838016663935 10.1177/1524838016663935 PMC5577387 27535921

[bibr19-08862605241253026] Hoi Nga Ng A. Weng Boey K. Wai Kwan C. Yim Fan Ho R. Yee Lin Ho D . (2022) Sexual self-concept and psychological functioning of women with a history of childhood sexual abuse in Hong Kong. International Journal of Sexual Health, 34(2), 177–196. 10.1080/19317611.2021.2022819 38596530 PMC10903682

[bibr20-08862605241253026] Hudson W. W. Harrison D. F. Crosscup P. C. (1981). A short-form scale to measure sexual discord in dyadic relationships. Journal of Sex Research, 17(2), 157–174.

[bibr21-08862605241253026] Jiwatram-Negrón T. Cheng S. Y. Wachter K. Mazzio A. K. Ward M. Reed L. Messing J. T. (2022). Examining associations between multiple types of IPV and adverse mental health among IPV survivors. Journal of Family Violence, 39, 177–191. 10.1007/s10896-022-00472-9

[bibr22-08862605241253026] Kamimura A. Nourian M. M. Assasnik N. Franchek-Roa K. (2016). Depression and intimate partner violence among college students in Iran. Asian Journal of Psychiatry, 23, 51–55. 10.1016/j.ajp.2016.07.014 27969079

[bibr23-08862605241253026] Katz J. Poleshuck E. L. Beach B. Olin R. (2017). Reproductive coercion by male sexual partners: Associations with partner violence and college women’s sexual health. Journal of Interpersonal Violence, 32(21), 3301–3320. 10.1177/0886260515597441 26246119 PMC7144871

[bibr24-08862605241253026] Lagdon S. Armour C. Stringer M. (2014). Adult experience of mental health outcomes as a result of intimate partner violence victimisation: A systematic review. European Journal of Psychotraumatology, 5(1), 24794. 10.3402/ejpt.v5.24794 PMC4163751 25279103

[bibr25-08862605241253026] Lévesque S. Rousseau C. Dumerchat M. (2021). Influence of the relational context on reproductive coercion and the associated consequences. Violence Against Women, 27(6–7), 828–850. https://doi.org/10.1177/1077801220917454 32469264 10.1177/1077801220917454

[bibr26-08862605241253026] Lévesque S. Rousseau C. (2019). Young women’s acknowledgment of reproductive coercion: A qualitative analysis. Journal of Interpersonal Violence, 36(15–16), NP8200–NP8223. 10.1177/0886260519842169 30973051

[bibr27-08862605241253026] Lovibond P. F. Lovibond S. H. (1993). Manual for the depression anxiety stress scales. Sydney Psychology Foundation.

[bibr28-08862605241253026] Loxton D. Powers J. Fitzgerald D. Forder P. Anderson A. Taft A. Hegarty K. (2013). The community composite abuse scale: Reliability and validity of a measure of intimate partner violence in a community survey from the ALSWH. Journal of Women’s Health, Issues & Care, 2(4), 1–7. 10.4172/2325-9795.1000115

[bibr29-08862605241253026] McCauley H. L. Falb K. L. Streich-Tilles T. Kpebo D. Gupta J. (2014). Mental health impacts of reproductive coercion among women in Côte D’ivoire. International Journal of Gynecology & Obstetrics, 127(1), 55–59. 10.1016/j.ijgo.2014.04.011 24952817 PMC5783185

[bibr30-08862605241253026] Miller E. Decker M. R. McCauley H. L. Tancredi D. J. Levenson R. R. Waldman J. Schoenwald P. Silverman J. G. (2010). Pregnancy coercion, intimate partner violence and unintended pregnancy. Contraception, 81(4), 316–322. 10.1016/j.contraception.2009.12.004 20227548 PMC2896047

[bibr31-08862605241253026] Mitchell E. Bennett L. R. (2020). Pressure and persuasion: Young Fijian women’s experiences of sexual and reproductive coercion in romantic relationships. Violence Against Women, 26(12–13), 1555–1573. 10.1177/1077801219882505 31663433

[bibr32-08862605241253026] Monteleone P. Mascagni G. Giannini A. Genazzani A. R. Simoncini T. (2018). Symptoms of menopause—global prevalence, physiology and implications. Nature Reviews Endocrinology, 14(4), 199–215. 10.1038/nrendo.2017.180 29393299

[bibr33-08862605241253026] Moulton J. E. Corona M. I. V. Vaughan C. Bohren M. A. (2021). Women’s perceptions and experiences of reproductive coercion and abuse: A qualitative evidence synthesis. PLoS One, 16(12), e0261551. 10.1371/journal.pone.0261551 PMC8691598 34932570

[bibr34-08862605241253026] Parliament of Australia. (2022, March 21). Identity matters: Sexual identity in Australia. https://www.aph.gov.au/About_Parliament/Parliamentary_departments/Parliamentary_Library/FlagPost/2022/March/Sexual_identity_in_Australia

[bibr35-08862605241253026] Pavot W. Diener E. (2008). The satisfaction with life scale and the emerging construct of life satisfaction. The Journal of Positive Psychology, 3(2), 137–152. 10.1080/17439760701756946

[bibr36-08862605241253026] Price E. Sharman L. S. Douglas H. A. Sheeran N. Dingle G. A. (2022). Experiences of reproductive coercion in Queensland women. Journal of Interpersonal Violence, 37(5–6), NP2823–NP2843. 10.1177/0886260519846851 31057040

[bibr37-08862605241253026] Rowlands S. Walker S. (2019). Reproductive control by others: means, perpetrators and effects. BMJ Sex & Reproductive Health, 45(1), 61–67. 10.1136/bmjsrh-2018-200156 30622127

[bibr38-08862605241253026] Scott J. G. Malacova E. Mathews B. Haslam D. M. Pacella R. Higgins D. J. Meinck F. Dunne M. P. Finkelhor D. Erskine H. E. Lawrence D. M. Thomas H. J. (2023). The association between child maltreatment and mental disorders in the Australian Child Maltreatment Study. Medical Journal of Australia, 218(Suppl 6), S26–S33. 10.5694/mja2.51870 PMC10952950 37004186

[bibr39-08862605241253026] Seth P. Wingood G. M. Robinson L. S. Raiford J. L. DiClemente R. J. (2015). Abuse impedes prevention: The intersection of intimate partner violence and HIV/STI risk among young African American women. AIDS and Behavior, 19(8), 1438–1445. 10.1007/s10461-014-0940-7 25399033 PMC4433610

[bibr40-08862605241253026] Sheeran N. Vallury K. Sharman L. S. Corbin B. Douglas H. Bernardino B. Hach M. Coombe L. Keramidopoulos S. Torres-Quiazon R. Tarzia L. (2022). Reproductive coercion and abuse among pregnancy counselling clients in Australia: Trends and directions. Reproductive Health, 19(1), 170. 10.1186/s12978-022-01479-7 35907880 PMC9338495

[bibr41-08862605241253026] Smylie L. Clarke B. Doherty M. Gahagan J. Numer M. Otis J. Smith G. McKay A. Soon C. (2013). The development and validation of sexual health indicators of Canadians aged 16–24 years. Public Health Reports, 128(Suppl 1), 53–61. 10.1177/00333549131282S106 PMC3562746 23450885

[bibr42-08862605241253026] Snell W.E. Jr. Fisher T.D. Waiters A.S. (1993). The multidimensional sexuality questionnaire: An objective self-report measure of psychological tendencies associated with human sexuality. Annals of Sex Research, 6, 27–55.

[bibr43-08862605241253026] Swan H. O’Connell D. J. (2012). The impact of intimate partner violence on women’s condom negotiation efficacy. Journal of Interpersonal Violence, 27(4), 775–792. 10.1177/0886260511423240 21987514 PMC4451787

[bibr44-08862605241253026] Tarzia L. Hegarty K. (2021). A conceptual re-evaluation of reproductive coercion: centring intent, fear and control. Reproductive Health, 18(1), 1–10. 10.1186/s12978-021-01143-6 33906687 PMC8077849

[bibr45-08862605241253026] Tarzia L. Douglas H. Sheeran N. (2022) Reproductive coercion and abuse against women from minority ethnic backgrounds: Views of service providers in Australia. Culture, Health & Sexuality, 24(4), 466–481, http://doi.org/10.1080/13691058.2020.1859617 10.1080/13691058.2020.1859617 33428538

[bibr46-08862605241253026] Weiss D. S. Mamar C. R. (1997). The impact of event scale revised. In Wilson J. P. Keane T. M. (Eds.), Assessing pychological trauma and PTSD: A practitioner’s handbook. (pp. 399–411). Guilford Press.

[bibr47-08862605241253026] World Health Organization. (2006). Defining sexual health: Report of a technical consultation on sexual health 28–31. https://www.who.int/teams/sexual-and-reproductive-health-and-research/key-areas-of-work/sexual-health/defining-sexual-health

[bibr48-08862605241253026] Yoshioka E. Palatino M. Nazareno J. Operario D. (2022). Intimate partner violence and sexual agency in a nationally representative sample of women and girls in the Philippines. Journal of Interpersonal Violence, 37(11–12), NP8867–NP8889. 10.1177/0886260520976208 33300443

